# Delayed reconfiguration of a non-emotional task set through reactivation of an emotional task set in task switching: an ageing study

**DOI:** 10.1080/02699931.2019.1567462

**Published:** 2019-01-17

**Authors:** Natalie Berger, Anne Richards, Eddy J. Davelaar

**Affiliations:** Department of Psychological Sciences, Birkbeck, University of London, London, UK

**Keywords:** Task switching, ageing, task relevance of emotion, emotional faces, arousal

## Abstract

In our everyday life, we frequently switch between different tasks, a faculty that changes with age. However, it is still not understood how emotion impacts on age-related changes in task switching. Using faces with emotional and neutral expressions, Experiment 1 investigated younger (*n* = 29; 18–38 years old) and older adults’ (*n* = 32; 61–80 years old) ability to switch between an emotional and a non-emotional task (i.e. responding to the face's expression vs. age). In Experiment 2, younger and older adults also viewed emotional and neutral faces, but switched between two non-emotional tasks (i.e. responding to the face's age vs. gender). Data from Experiment 1 demonstrated that switching from an emotional to a non-emotional task was slower when the expression of the new face was emotional rather than neutral. This impairment was observed in both age groups. In contrast, Experiment 2 revealed that neither younger nor older adults were affected by block-wise irrelevant emotion when switching between two non-emotional tasks. Overall, the findings suggest that task-irrelevant emotion can impair task switching through reactivation of the competing emotional task set. They also suggest that this effect and the ability to shield task-switching performance from block-wise irrelevant emotion are preserved in ageing.

Switching between different tasks is so common in everyday life that we are often not even aware that we perform task switching. For instance, putting together a shopping list requires switching between thinking of ingredients you need, checking for them in cupboards, and writing them down. The ability to switch between tasks is a core executive function that plays an important role in everyday functioning (Miyake & Friedman, 2012; Miyake et al., 2000) and given evidence that emotion can affect executive functions (Pessoa, 2009, 2015, 2017), research has also started to assess interactions between emotion and task switching (Aboulafia-Brakha, Manuel, & Ptak, 2016; de Vries & Geurts, 2012; Gul & Khan, 2014; Johnson, 2009; Paulitzki, Risko, Oakman, & Stolz, 2008; Piguet et al., 2013, 2016; Reeck & Egner, 2015). However, emotional task switching in ageing has not been investigated despite evidence of age-related changes in both executive functions (Babcock & Salthouse, 1990; Braver & West, 2008; MacPherson, Phillips, & Della Sala, 2002; Reuter-Lorenz & Sylvester, 2005; Salthouse, 1990, 1991; Zelazo, Craik, & Booth, 2004) and in emotional functioning (Blanchard-Fields, 2007; Blanchard-Fields, Mienaltowski, & Seay, 2007; Carstensen, Pasupathi, Mayr, & Nesselroade, 2000; Gross et al., 1997; Larcom & Isaacowitz, 2009; Scheibe & Blanchard-Fields, 2009). The present study was conducted to close this empirical gap by comparing emotional task switching in younger and older adults.

## Task switching and the role of emotions

Switching between tasks is usually associated with slower reaction times (RTs) and more errors compared to repeating a task, which is referred to as the switch cost (Kiesel et al., 2010; Mayr & Kliegl, 2000; Meiran, Chorev, & Sapir, 2000; Monsell, 2003; Rogers & Monsell, 1995; Wylie & Allport, 2000). Multiple processes are thought to contribute to switch costs, including task set reconfiguration (Monsell, 2003; Rogers & Monsell, 1995), which relates to the engagement with the new task, as well as proactive interference from the no longer relevant task on switch trials (Allport, Styles, & Hsieh, 1994; Wylie & Allport, 2000), which relates to the disengagement from the previous task. Studies investigating the effects of emotion on task switching have primarily focused on comparing the switch costs associated with switching between an emotional and a non-emotional task, but the results are equivocal. Some studies showed that switching away from an emotional to a non-emotional task took longer than vice versa (Johnson, 2009; Paulitzki et al., 2008), others reported evidence suggesting that non-emotional tasks are more difficult to switch to and away from relative to emotional tasks (Reeck & Egner, 2015; Schuch, Werheid, & Koch, 2012), whereas no differences in switch costs between emotional and non-emotional tasks were found in another study (Gul & Khan, 2014). Given these mixed results, it is still an open question how emotion affects task switching and if it affects sub-processes such as task set reconfiguration or proactive interference differently.

It should be noted that these studies varied in the applied methodology, which could have contributed to inconsistent findings. Studies reporting higher costs for switches from emotional to non-emotional tasks than vice versa (Johnson, 2009; Paulitzki et al., 2008) have used tasks that varied in perceptual salience and size (e.g. response to a large, real-life picture of a spider in the emotional task vs. response to a small number placed over the spider's back in the neutral task). The salience of emotional stimuli due to size alone could have contributed to greater switch costs from emotional to non-emotional tasks than vice versa. Other studies (Gul & Khan, 2014; Reeck & Egner, 2015; Schuch et al., 2012) used tasks with perceptually balanced stimuli by asking participants to respond to the expression of a face in the emotional task and to the gender or age of a face in non-emotional task. However, emotional stimuli were either used on every trial (Reeck & Egner, 2015; Schuch et al., 2012), or no distinction between emotional (i.e. happy) and neutral trials was made in the analysis (Gul & Khan, 2014). Given that continuous presentation of emotional material can lead to habituation (Breiter et al., 1996; Phan, Liberzon, Welsh, Britton, & Taylor, 2003; Zald, 2003), this might have facilitated switching to a non-emotional task set in studies with emotional material only. Moreover, the notion that faster switching to a non-emotional than to an emotional task was due to the inhibition of the more dominant emotional task set (Reeck & Egner, 2015) cannot be tested without a comparison between switches to emotional and neutral items in the non-emotional task. It is also possible that emotion affected sub-processes such as reconfiguration and interference differently despite no overall differences in switch costs between emotional and non-emotional tasks sets in Gul and Khan’s (2014) study. Additionally, Gul and Khan (2014) used happy faces, whereas only negative (e.g. threat-related pictures) or a mix of positive and negative items (e.g. happy and angry faces) were used as emotional material in other studies (Johnson, 2009; Paulitzki et al., 2008; Reeck & Egner, 2015; Schuch et al., 2012). This might have contributed to different patterns of results. Finally, repeat and switch trials were not always balanced between competing tasks in previous research, which might have affected the results. For instance, (Johnson, 2009) used three times more repetition trials for the emotion relative to the non-emotional task, which might have affected participants’ ability to disengage from the emotional task and/or to reconfigure the non-emotional task.

The role of repeat trials in task switching indeed deserves further attention as many previous studies have used switch cost as the dependent variable. However, subtracting the latencies of repeat from those of switch trials makes it impossible to disentangle whether the difference is driven by (faster) repeat or (slower) switch responses. As the experimental design of previous studies allowed for response repetition on repeat trials, this might have contributed to particularly fast repeat responses on average and thus, to higher switch costs. Furthermore, processing of emotional items is known to be particularly efficient (e.g. Phelps & LeDoux, 2005; Phelps, Ling, & Carrasco, 2006), and thus, it is possible that the speed-up effect for repeat trials was particularly pronounced for responses to emotional (e.g. expression of a face) rather than non-emotional item features (e.g. gender of a face). In sum, it is possible that there were differences between switch costs for emotional and non-emotional tasks due to effects on repeat rather than on switch trials.

## Task switching between emotional and non-emotional tasks in ageing

Research suggests that ageing is associated with reduced ability to maintain and schedule two different tasks in working memory (Reimers & Maylor, 2005; Verhaeghen & Cerella, 2002; Wasylyshyn, Verhaeghen, & Sliwinski, 2011), which is labelled *global* task switching and examined by comparing performance in single-task blocks with performance in blocks with multiple tasks requiring task switching. In contrast, no general impairments were found for the ability to activate and deactivate task sets flexibly, which is labelled *local* task switching and assessed by comparing performance on switch and repeat trials (Reimers & Maylor, 2005; Verhaeghen & Cerella, 2002; Wasylyshyn et al., 2011). However, age-related differences in local task switching were found to emerge, for instance, when the number of tasks were increased from two to four and when unpredictable switching was required (Kray, Li, & Lindenberger, 2002). The present study was conducted to extend previous research by investigating whether local task switching is also affected differently by emotion in younger and older adults.

According to previous research, older adults tend to direct their attention to positive and away from negative material (for reviews, see Reed & Carstensen, 2012; Scheibe & Carstensen, 2010), which could affect their ability to engage with or disengage from emotional items during task switching. The socioemotional selectivity theory (SST; Carstensen, 1993) suggests that older adults focus on positive material to enhance their well-being, which results in the positivity effect in ageing. A different view comes from the dynamic integration theory (see Labouvie-Vief, 2003, 2009 for reviews), which links the positivity effect with age-related cognitive decline by stating that older adults tend to avoid cognitively more demanding negative affect. Although these theories suggest different underlying mechanisms for the positivity effect in ageing, both would predict that older adults engage more readily with positive than with negative material and disengage more readily from negative than from positive material. In a task-switching task, this could lead to age-related changes in the ability to switch to or away from positive and negative items despite unimpaired local task switching in ageing. To assess the effects of emotion on the reconfiguration of the new task and the interference from the previous task, the valences of the target item and the previous item were considered in Experiment 1 and the following hypotheses were tested:

### Reconfiguration account

If emotion affects engagement with the new task, target emotion is expected to influence task switching. Given that emotional material is more salient than neutral material, emotional items will be easier to engage with relative to neutral items. Given older adults’ preference for positive material, it is expected that older but not younger adults will be faster to engage with positive compared to neutral or negative items.

### Interference account

If emotion affects interference from the previous task, previous emotion is expected to influence task switching. It is possible that emotional items will be more difficult to disengage from relative to neutral items due to their enhanced salience. Given older adults’ preference for positive material, it can be expected that older but not younger adults will be slower to disengage from positive relative to neutral or negative items.

## Methods

### Participants

Thirty-two younger (18–38 years old) and 32 older adults (61–80 years old) participated in Experiment 1 (see Table 1 for participant characteristics). The sample size was determined on the basis of related work with similar experimental conditions (Gul & Khan, 2014; Reeck & Egner, 2015). Three younger participants were excluded from the analysis due to failure to follow instructions, resulting in a final sample of 29 younger and 32 older adults. Younger adults were undergraduate and postgraduate students at Birkbeck, University of London, and older adults were community-dwelling volunteers recruited from the University of the Third Age in London. All participants received a small fee for taking part. They reported to be in good health and to have normal or corrected-to-normal vision and hearing. They were pre-screened for psychiatric disorders and a history of neurological disorders. Older adults had a score of 27 or above on the MMSE (Folstein, Folstein, & McHugh, 1975).

**Table 1.** Participant characteristics.

	Younger adults		Older adults		Group difference	
Variable	*M*	*SD*	*M*	*SD*	*t*	*p*
Age	25.34	5.86	70.93	5.25	−31.50	<.001
Gender (male:female)	14:15		9:23			
Years of education	16.86	3.16	15.73	2.94	1.42	.160
NART Verbal IQ	106.48	7.74	117.30	5.45	−6.80	<.001
Digit Symbol Test	70.52	12.80	52.63	12.72	5.38	<.001
BDI II	4.96	5.32	4.00	4.14	.77	.446
STAI Trait Anxiety	37.82	11.78	32.87	8.61	1.84	.072
MMSE			29.17	.91		

Note: NART = The National Adult Reading Test, BDI II = Beck Depression Inventory II, STAI = State-Trait Anxiety Inventory, MMSE = Mini-Mental State Examination.

As can be seen in Table 1, older adults had better verbal knowledge than younger adults as assessed with the NART (Nelson & Willison, 1991) and scored lower on the Digit Symbol Substitution Test (Wechsler, 1955) suggesting slower processing speed than in younger adults. These results of better vocabulary knowledge (Alwin & McCammon, 2001; Bowles, Grimm, & McArdle, 2005) and slower processing speed (Salthouse, 1996, 2000) in older than younger adults are consistent with typical findings in ageing research. No further differences were observed between the two age groups. This study was carried out in accordance with the recommendations of the ethics board of Birkbeck, University of London. All participants gave written informed consent in accordance with the Declaration of Helsinki.

### Materials

Stimuli consisted of 48 images of faces from the FACES database (Ebner, Riediger, & Lindenberger, 2010), a validated set of colour photographs of naturalistic, front-facing faces of different ages. In a preliminary rating study (Berger, Richards, & Davelaar, 2017), ten younger (21–32 years old; *M* = 27.80, *SD* = 3.12) and ten older adults (66–76 years old; *M* = 71.27, *SD* = 3.13) rated the valence and arousal of 234 preselected faces. Sixteen happy, 16 angry and 16 neutral expressions with the highest agreement between younger and older raters were then selected for the main experiment. The age group (younger, older) and sex (male, female) of the faces were balanced in each emotion category and each picture showed a unique individual. For counterbalancing purposes, two face sets with similar arousal and valence levels (all *ts*(19) < 1.30, *ps* < .208) were created.

### Procedure

After giving informed consent, participants completed a demographic questionnaire and were seated in front of a computer screen. A visual acuity test (Bach, 1996) was conducted at a distance of 65 cm to ensure that vision was in the normal range. Participants were then instructed to remain at this distance to the screen and to perform the computerised task, which was prepared and presented using E-Prime Version 2.0.10.353 (Schneider, Eschman, & Zuccolotto, 2002) on a 24-inch computer screen with a resolution of 1920 × 1200 pixels. To allow for binary responses, the task consisted of two blocks: one comprising 16 unique faces of younger and older adults with neutral and happy expressions and one comprising 16 unique faces of younger and older adults with neutral and angry expressions. In each block, 160 faces were presented in a random order: 40 were young and neutral, 40 were young and emotional, 40 were old and neutral and 40 were old and emotional. Each face was viewed approximately 10 times per block. Faces from different subsets were presented in each block to ensure that neutral faces, which were included in both blocks, were different across blocks. The order of the blocks was counterbalanced across participants. Participants had the option to take short breaks every 40 trials, resulting in three breaks per block and a break between blocks. The task was preceded by 16 unscored practice trials.

**Figure 1.**
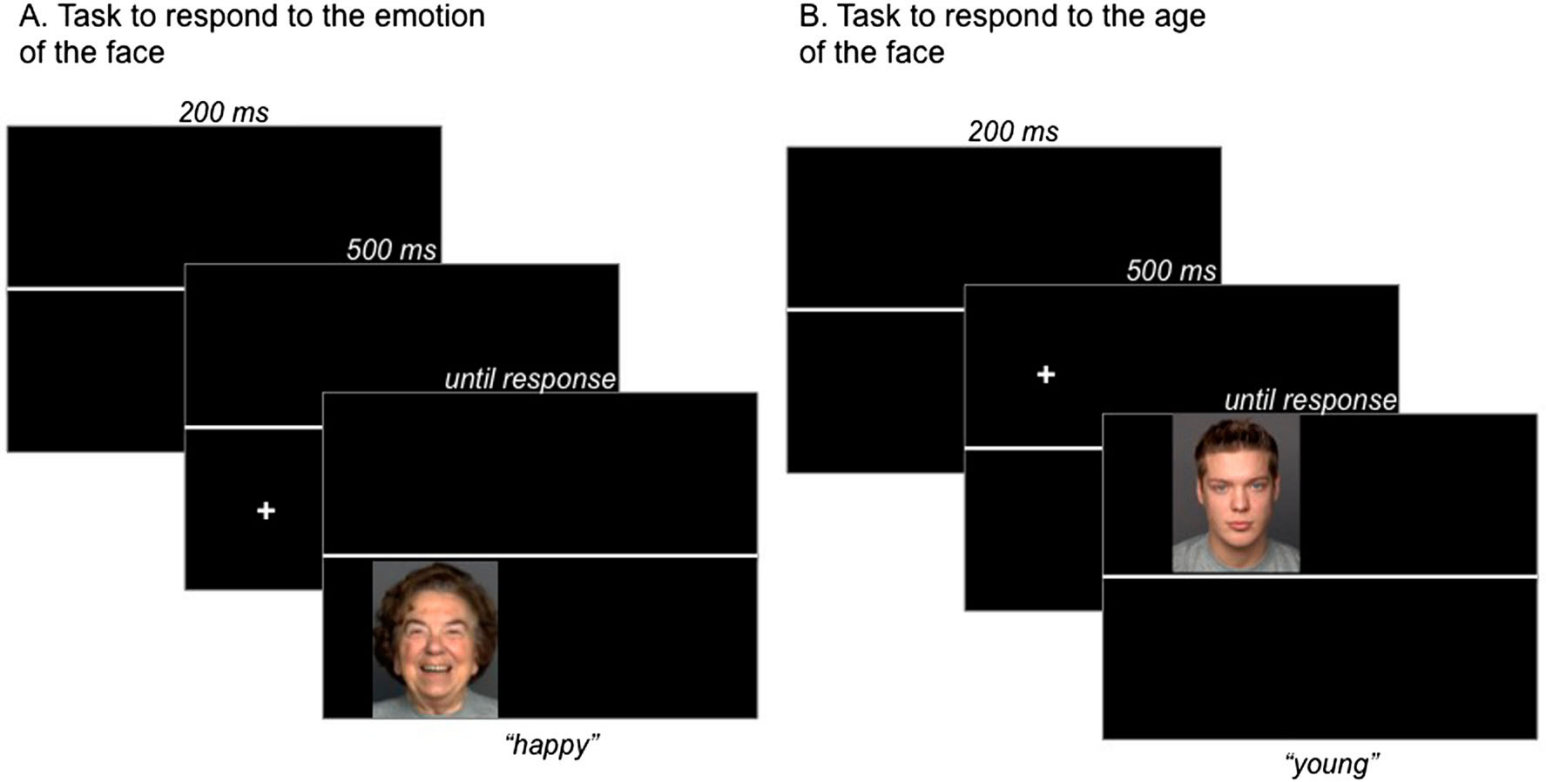
Examples of trials with the instructions to respond to the emotion of the face if it is presented below the horizontal line (A) or to the age of the face if it is presented above the horizontal line (B) in Experiment 1. As faces were presented clockwise, the examples show a repeat trial in the emotion condition and a switch trial in the age condition.

In each trial, participants viewed one face at a time that was presented in the left top quarter, the right top quarter, the bottom right quarter or the bottom left quarter of the screen. A horizontal line separated the top and bottom of the screen and was visible for the duration of the whole block. Face presentation, which was preceded by a fixation cross for 500 ms in the relevant location, always started in the left top quarter and continued clockwise before starting again in the left top quarter. Participants were instructed to respond to the age of the face by pressing one of two buttons (“young” vs. “old”) when the face was presented above (or below) the horizontal line and to the emotion of the face by pressing one of two buttons (“neutral” vs. “emotional”) when the face was presented below (or above) a horizontal line. Button presses initiated the next trial after the presentation of a blank screen (with the horizontal line) for 200 ms. Task assignment to the top or bottom half of the screen was counterbalanced across participants, as was the labelling of the keys. Task switching was associated with hand switching: responses to the task on the top half of the screen were assigned to the left hand and responses to the task on the bottom half of the screen were assigned to the right hand to avoid conflict at the response level. On a regular PC computer keyboard, the buttons “S” and “D” as well as “K” and “L” were used. An example of a trial sequence is presented in Figure 1.

After the computer task, participants completed the Digit Symbol Substitution Test (Wechsler, 1955), the NART (Nelson & Willison, 1991), the Beck Depression Inventory (BDI-II; Beck, Steer, & Carbin, 1988) and the A-Trait version of the State-Trait Anxiety Inventory (STAI; Spielberger, Gorsuch, Lushene, Vagg, & Jacobs, 1983). Older adults additionally completed the MMSE (Folstein et al., 1975). Participants were debriefed at the end of the session, which lasted 60–75 min.

### Design and statistical analysis

Responses and RTs were recorded for each trial. For the analyses of RTs, any RTs faster than 200 ms or 2.5 standard deviations above or below the age group's mean RTs were excluded, resulting in an exclusion of an average of 2.86% of trials for younger adults and 2.51% for older adults. Accuracy and median RTs for correct trials were calculated for each condition. As explained further above, the hypotheses related to the effects of emotion and age on switch trials and thus, only analyses for switch trials are reported below. Separate analyses for repeat trials are reported in the Supplemental Material.

Statistical analysis of the data was conducted with SPSS 22 (IBM Corp., Armonk, NY). Accuracy and RTs in the happy vs. neutral and the angry vs. neutral blocks were analysed separately. Data were analysed by a 2 × 2 × 2 × 2 mixed factors ANOVA with the within-subjects factors task (age vs. emotion), target emotion (happy/angry vs. neutral) and previous emotion (happy/angry vs. neutral) and the between-subjects factor age (younger vs. older). Post-hoc *t-*tests with a Bonferroni adjustment to the 5% alpha level were performed to follow up significant interactions. Due to significant differences in the two age groups’ verbal knowledge and processing speed, all analyses were repeated with NART verbal IQ and Digit Symbol as centred covariates. The results with age as a factor reported here were qualitatively the same and significant in the analysis including covariates unless stated otherwise.

## Results

### Happy vs. neutral faces

#### Accuracy

**Figure 2.**
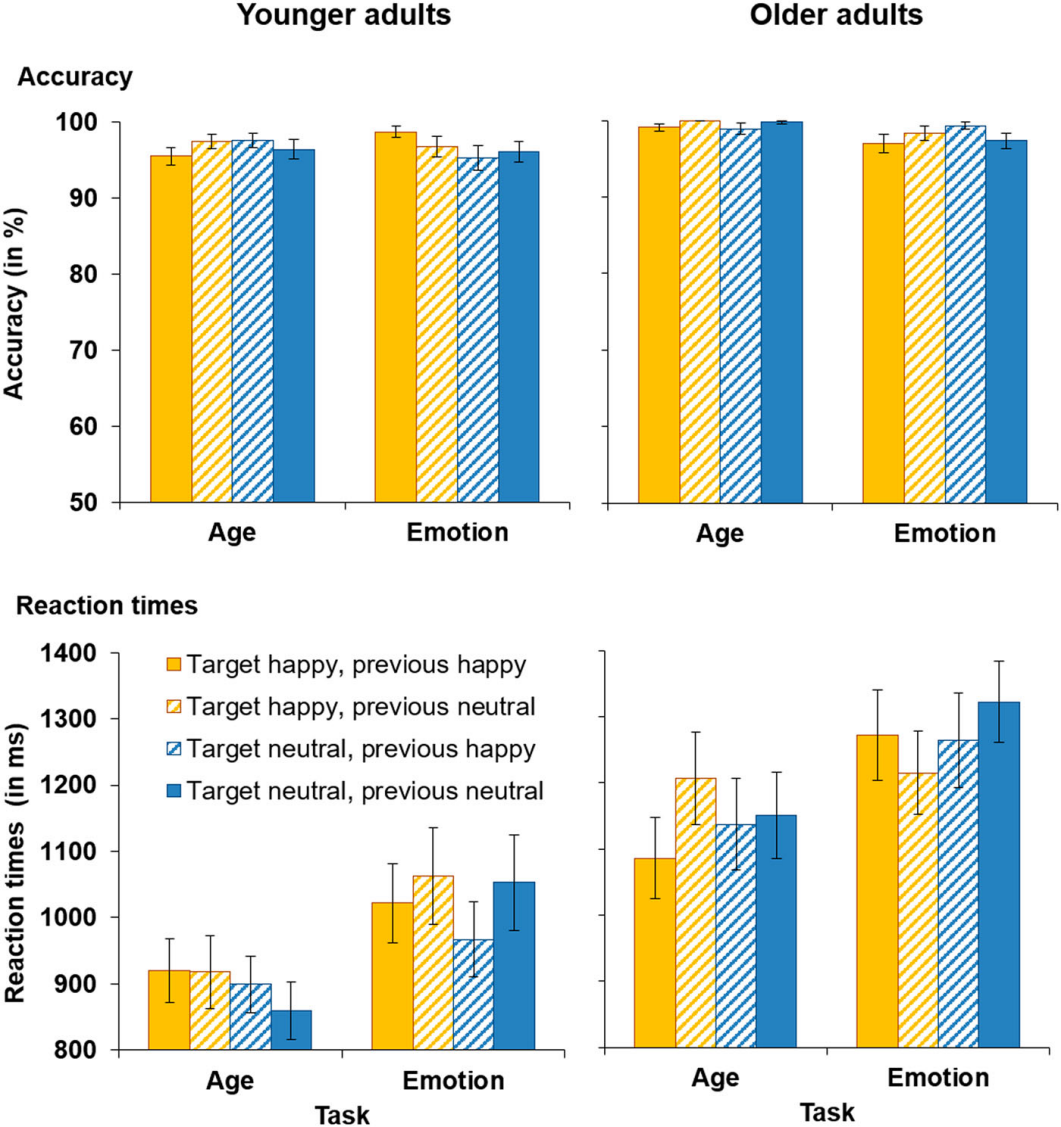
Accuracy (upper panels) and RTs for correct responses (lower panels) in younger (left-hand panels) and older adults (right-hand panels) as a function of target emotion and previous emotion in Experiment 1. Participants switched between the age task (with task-irrelevant emotion) and the emotion task (with task-relevant emotion). This figure shows data from the happy vs. neutral task block. Only switch trials are presented in this figure. Error bars represent SEM.

Accuracy for performance in the happy vs. neutral task block in younger and older adults is presented in Figure 2 (top panels). The four-way omnibus ANOVA revealed a main effect of age, *F*(1, 59) = 11.12, *MSE* = .005, *p* = .001, partial *η^2^* = .16, as older adults were more accurate (*M* = 98.7%, *SD* = 2.1%) than younger adults (*M* = 96.7%, *SD* = 2.8%). No further significant main effects or interactions were observed for accuracy for happy vs. neutral faces (all *F*s < 3.00).

#### Reaction times

RTs for performance in the happy vs. neutral task block in younger and older adults are presented in Figure 2 (bottom panels). The four-way omnibus ANOVA yielded a main effect of task, *F*(1, 59) = 17.40, *MSE* = 89530, *p* < .001, partial *η^2^* = .23, with overall slower RTs in the emotion task (*M* = 1153 ms, *SD* = 343 ms) compared to the age task (*M* = 1041 ms, *SD* = 323 ms). This main effect was qualified by a task × target emotion interaction, *F*(1, 59) = 4.14, *MSE* = 21865, *p* = .046, partial *η^2^* = .07. Follow-up *t*-tests revealed that in the age task, RTs were slower for happy targets (*M* = 1064 ms, *SD* = 329 ms) than for neutral targets (*M* = 1018 ms, *SD* = 332 ms), *t*(60) = 2.50, *p* = .015. In the emotion task, there was no significant difference between RTs for neutral or happy targets (*p* = .650). Finally, there was also a main effect of age, *F*(1, 59) = 11.81, *MSE* = 676967, *p* = .001, partial *η^2^* = .17, as older adults were overall slower (*M* = 1219 ms, *SD* = 329 ms) than younger adults (*M* = 963 ms, *SD* = 242 ms). No further significant main effects or interactions were observed for RTs to happy vs. neutral faces (all *F*s < 3.10).

### Angry vs. neutral faces

#### Accuracy

**Figure 3.**
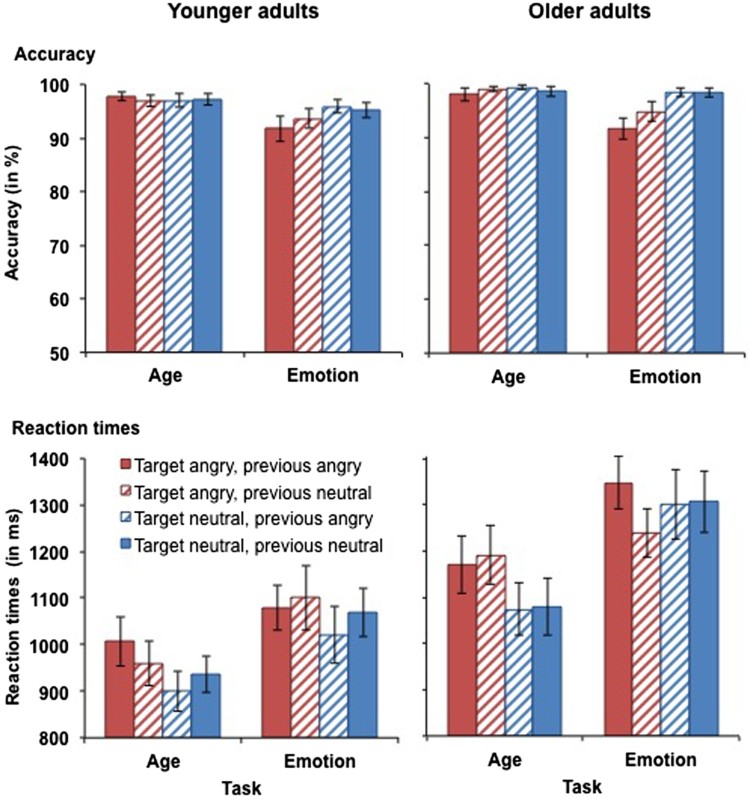
Accuracy (upper panel) and RTs for correct responses (lower panel) in younger (left-hand panel) and older adults (right-hand panel) as a function of target emotion and previous emotion in Experiment 1. Participants switched between the age task (with task-irrelevant emotion) and the emotion task (with task-relevant emotion). This figure shows data from the angry vs. neutral task block. Only switch trials are presented in this figure. Error bars represent SEM.

Accuracy scores for performance in the angry vs. neutral task block in younger and older adults are presented in Figure 3 (top panels). The four-way omnibus ANOVA yielded a main effect of task, *F*(1, 59) = 14.99, *MSE* = .007, *p* < .001, partial *η^2^* = .20, as accuracy scores were higher in the age task (*M* = 98.0%, *SD* = 3.0%) than in the emotion task (*M* = 95.0%, *SD* = 5.8%). There was also a main effect of target emotion, *F*(1, 59) = 7.58, *MSE* = .006, *p* = .008, partial *η^2^* = .11, with lower accuracy scores for angry targets (*M* = 95.5%, *SD* = 5.6%) than for neutral targets (*M* = 97.6%, *SD* = 3.2%). This main effect was qualified by a task × target emotion interaction, *F*(1, 59) = 7.58, *MSE* = .006, *p* = .008, partial *η^2^* = .11. Follow-up *t*-tests revealed that in the emotion task, accuracy was lower for angry targets (*M* = 93.0%, *SD* = 9.7%) than for neutral targets (*M* = 97.0%, *SD* = 4.3%), *t*(60) = 3.32, *p* = .002, whereas in the age task, accuracy scores for neutral and angry targets did not differ (*p* = .909). No further significant main effects or interaction were observed for accuracy scores for angry vs. neutral faces (all *F*s < 3.20).

#### Reaction times

RTs for performance in the angry vs. neutral task block in younger and older adults are presented in Figure 3 (bottom panels). The four-way omnibus ANOVA yielded a main effect of task, *F*(1, 59) = 50.09, *MSE* = 49662, *p* < .001, partial *η^2^* = .46, as participants were slower in the emotion task (*M* = 1188 ms, *SD* = 315 ms) than in the age task (*M* = 1044 ms, *SD* = 287 ms). There was also a main effect of target emotion, *F*(1, 59) = 8.15, *MSE* = 38061, *p* = .006, partial *η^2^* = .12, with slower RTs for angry targets (*M* = 1141 ms, *SD* = 291 ms) relative to neutral targets (*M* = 1091 ms, *SD* = 307 ms). This main effect was qualified by a task × target emotion interaction, *F*(1, 59) = 4.18, *MSE* = 32855, *p* = .045, partial *η^2^* = .07. Separate analyses for the age task and the emotion task revealed that in the age task, RTs were slower for angry targets (*M* = 1087 ms, *SD* = 314 ms) than for neutral targets (*M* = 1002 ms, *SD* = 283 ms), *t*(60) = 4.08, *p* < .001. In contrast, there was no significant difference in RTs for neutral and angry targets in the emotion task (*p* = .567). There was also a main effect of age, *F*(1, 59) = 8.43, *MSE* = 601596, *p* = .005, partial *η^2^* = .13. However, this effect became non-significant (*p* = .102) when processing speed was included as a covariate in the analysis. No further significant main effects or interactions were observed for RTs for angry vs. neutral faces (all *F*s < 3.40).

## Discussion

The aim of Experiment 1 was to investigate the effects of emotion on switching between emotional and non-emotional tasks in younger and older adults. The main finding was that both age groups were slower when switching to emotional rather than neutral faces in the non-emotional age task, whereas no differences in RTs were observed for switches to neutral and emotional faces in the emotional expression task. Moreover, although the emotion of the target, which they had to switch to, modulated responses in both age groups, the emotion of the item, which they had to switch away from, did not. Lastly, there were no age-related differences in task switching besides evidence of a speed-accuracy trade-off in older adults.

The finding that participants were slower when having to switch to an emotional face in the age task suggests that trial-irrelevant emotion was processed and impaired performance in both age groups. One explanation for this effect could be that having just processed and responded to emotional information in the emotional task made it difficult for participants to switch to the non-emotional task (option 1). This would be in accordance with the suggestion that proactive interference from the no longer relevant task affects task switching (Allport et al., 1994; Wylie & Allport, 2000). Another explanation (option 2) could be that it simply took longer to identify a face's age if the face was emotional rather than neutral, as evidence suggests that emotional expressions can affect age ratings (Ganel, 2015; Voelkle, Ebner, Lindenberger, & Riediger, 2012). Alternatively, it is possible that emotional items triggered trial-irrelevant emotional processing either in an automatic fashion (option 3) or through reactivation of the competing emotional task set (option 4). Exogenous factors may play an important part in task switching through cueing of task sets (Kiesel et al., 2010; Rogers & Monsell, 1995; Rubin & Koch, 2006). Thus, it is possible that emotional items slowed down responses in the non-emotional task set relative to neutral items as they cued the currently irrelevant emotional task set.

Repeat trials were analysed (see Supplemental Material) to rule out some of these explanations. The analyses showed that RTs were slower for emotional compared to neutral target items for repeat trials in the age task just as it was the case for switch trials. Although this does not allow evaluating the validity of options 2, 3, and 4, it helps to eliminate option 1, according to which RTs for emotional trials were slower relative to neutral trials due to the difficulty to disengage from the emotion task of the previous trial. Given that RTs for repeat trials were faster than for switch trials, it can be assumed that participants were (at least to some degree) able to reconfigure the new non-emotional task set. Thus, it seems unlikely that the difficulty to disengage from the previously performed emotion task was responsible for the similar pattern of longer RTs for emotional relative to neutral trials in the age task for both switch and repeat trials. Instead, it appears that emotion cues were still effective in the non-emotional task even on repeat trials. To further explore the remaining explanations for a slowdown in switches to emotional relative to neutral items in the non-emotional task, Experiment 2 was conducted.

## Experiment 2

In Experiment 1, participants had to switch constantly between an emotional and a non-emotional task. As emotion was relevant for one of the competing tasks, it is possible that it affected performance in the non-emotional task due to its relevance for the task block (which included both the emotional and the non-emotional tasks). In other words, trial-irrelevant emotion affected performance as it was block-relevant. Alternatively, emotion might have been processed in an automatic way due to the salience of emotion (e.g. Phelps & LeDoux, 2005; Phelps et al., 2006) or the effects were simply due to the difficulties of identifying the emotional face's age (Ganel, 2015; Voelkle et al., 2012).

Although switching between emotional and non-emotional tasks has been investigated before (Aboulafia-Brakha et al., 2016; de Vries & Geurts, 2012; Gul & Khan, 2014; Johnson, 2009; Paulitzki et al., 2008; Piguet et al., 2016; Piguet et al., 2013; Reeck & Egner, 2015; Schuch et al., 2012), so far, none of the studies has assessed the effect of trial-irrelevant emotion on performance in a non-emotional task set. It has neither been investigated whether emotion has to be relevant for at least one task in order to affect switching or whether it does so even if it is block-wise irrelevant. The present experiment set out to close this empirical gap by investigating how switching between two non-emotional task sets is affected by block-wise irrelevant emotion.

In Experiment 2, the two tasks were identifying the age or gender of a face and participants had to switch between these two non-emotional tasks in the presence of happy, neutral and angry expressions. By introducing two non-emotional tasks with block-wise irrelevant emotion, this experiment was designed to distinguish between the following explanations for longer RTs in response to emotional relative to neutral items in the non-emotional task in Experiment 1: If longer RTs for emotional rather than neutral stimuli in the age task were due to difficulties to identify the face's age in emotional faces, we would expect to find this pattern for the age but not the gender task. Alternatively, if emotion affected task switching irrespective of its relevance for either of the two non-emotional tasks automatically, we would expect to find longer RTs for emotional than neutral faces in the age and in the gender task. However, if emotion affected performance in the non-emotional task due to reactivation of the competing emotional task through emotional cues, we would not expect to find an effect of emotion on task switching performance in the age task in the present experiment.

### Methods

#### Participants

Participants from Experiment 1 also took part in the present experiment in a single session. Half of participants started with the task in Experiment 1, whereas the other half started with the task in Experiment 2. Participants excluded from the analysis in Experiment 1 were also excluded from the analysis in Experiment 2.

#### Materials

The same stimuli as in Experiment 1 were used.

#### Procedure

The procedure and counterbalancing were identical to the procedure for Experiment 1. The only difference was that participants were instructed to respond to the age of the face by pressing one of two buttons (“young” vs. “old”) when the face was presented above (or below) the horizontal line and to the gender of the face by pressing one of two buttons (“male” vs. “female”) when the face was presented below (or above) the horizontal line.

#### Design and statistical analysis

Responses and RTs were recorded for each trial and the same exclusion criteria were applied as in Experiment 1, resulting in an exclusion of 2.28% of data points in the younger age group and 2.65% in the older age group. Accuracy and median RTs for correct trials were calculated for each condition. As in Experiment 1, only analyses for switch trials are reported below. Analyses for repeat trials are reported in the Supplemental Material. As in Experiment 1, accuracy and RTs were analysed separately for the happy vs. neutral and the angry vs. neutral blocks. Accuracy and RTs were analysed by 2 × 2 × 2 × 2 mixed factors ANOVA including the within-subjects factors task (age vs. gender), target emotion (happy/angry vs. neutral) and previous emotion (happy/angry vs. neutral) as well as the between-subjects factor age (younger vs. older). Post-hoc *t-*tests with a Bonferroni adjustment to the 5% alpha level were performed to follow up significant main effects and interactions. Due to significant differences in the two age groups’ verbal knowledge and processing speed, all analyses were repeated with NART verbal IQ and Digit Symbol as centred covariates as in Experiment 1. The results with age as a factor reported here were qualitatively the same and significant in the analysis including covariates unless stated otherwise. It should also be noted that the order of experiments interacted with task and with target emotion in the analysis of accuracy scores in Experiment 1 as well as with task in the analysis of RTs in Experiment 2 (see Supplemental Material for statistical analyses). Due to these interactions, all analyses reported in this paper were repeated with order of experiments as a covariate. The results reported here were qualitatively the same and significant in the analysis including order of experiments as a covariate.

### Results

#### Happy vs. neutral faces

**Figure 4.**
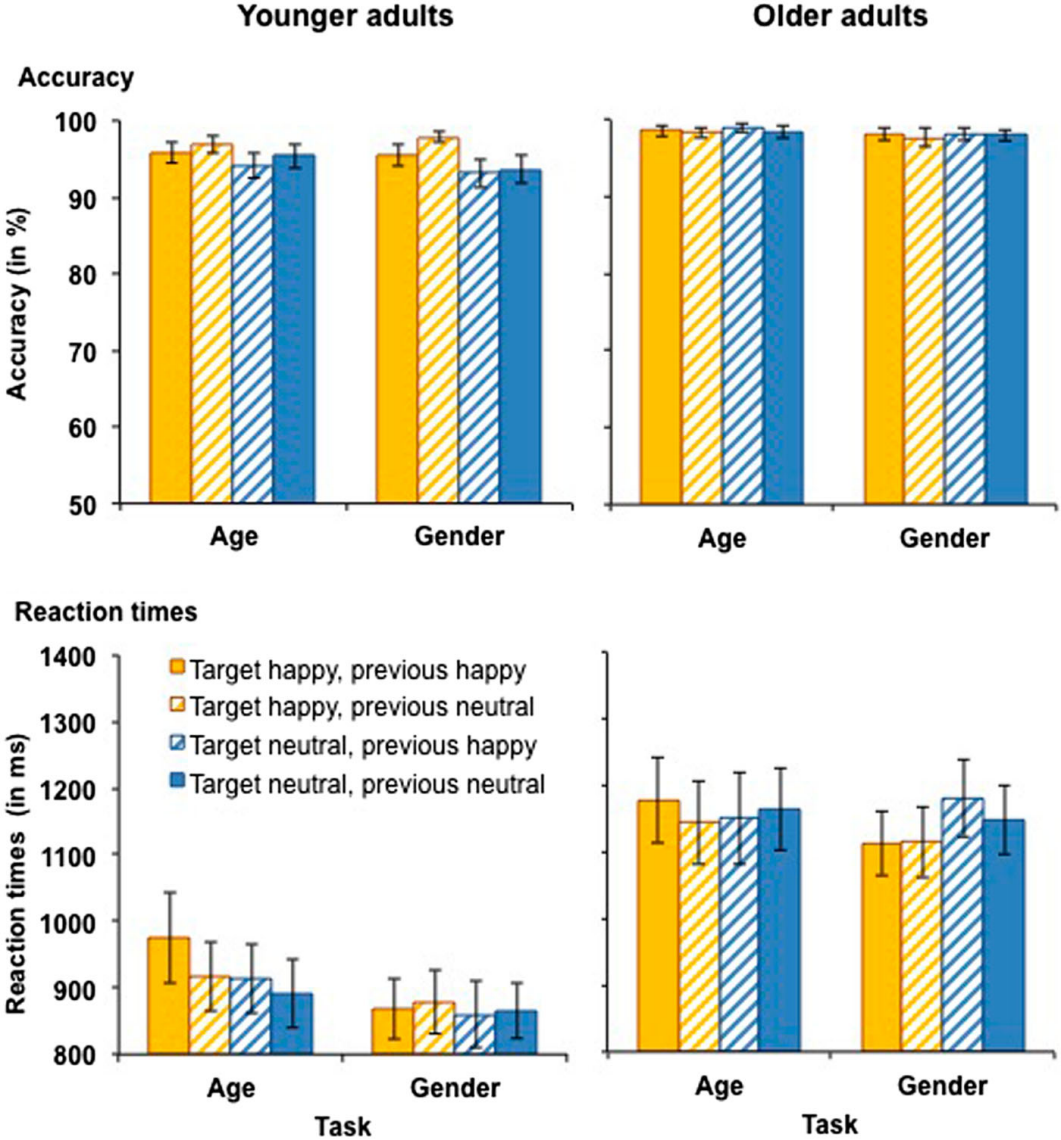
Accuracy (upper panel) and RTs for correct responses (lower panel) in younger (left-hand panel) and older adults (right-hand panel) as a function of target emotion and previous emotion in Experiment 2. Participants switched between the age and the gender tasks with block-wise irrelevant emotion. This figure shows data from the happy vs. neutral task block. Only switch trials are presented. Error bars represent SEM.

***Accuracy.*** Accuracy scores for performance in the happy vs. neutral task block in younger and older adults are presented in Figure 4 (top panels). The analysis of accuracy scores yielded a significant main effect of target emotion, *F*(1, 59) = 4.67, *MSE* = .003, *p* = .035, partial *η^2^* = .07, as accuracy was higher for happy (*M* = 97.4%, *SD* = 3.4%) than for neutral trials (*M* = 96.4%, *SD* = 4.7%). This main effect was qualified by a target emotion × age interaction, *F*(1, 59) = 6.51, *MSE* = .002, *p* = .013, partial *η^2^* = .10. Follow-up *t*-tests revealed that in younger adults, accuracy was higher for trials with happy faces (*M* = 96.6%, *SD* = 4.3%) than with neutral faces (*M* = 94.1%, *SD* = 5.4%), *t*(28) = 2.64, *p* = .013, whereas in older adults, there was no difference in accuracy for trials with happy and neutral faces (*p* = .699). There was also a main effect of age, *F*(1, 59) = 12.82, *MSE* = .004, *p* = .001, partial *η^2^* = .18, as accuracy was higher in older adults (*M* = 98.3%, *SD* = 2.0%) than in younger adults (*M* = 95.3%, *SD* = 4.2%). No further significant main effects or interactions were observed for accuracy scores for happy vs. neutral faces.

***Reaction times.*** RTs for performance in the happy vs. neutral task block in younger and older adults are presented in Figure 4 (bottom panels). The analysis revealed a main effect of task, *F*(1, 59) = 4.49, *MSE* = 38279, *p* = .038, partial *η^2^* = .07, as RTs were longer in the age task (*M* = 1047 ms, *SD* = 321 ms) than in the gender task (*M* = 1011 ms, *SD* = 286 ms). There was also a main effect of age, *F*(1, 59) = 13.42, *MSE* = 581514, *p* = .001, partial *η^2^* = .19, driven by slower RTs in older (*M* = 1115 ms, *SD* = 286 ms) than in younger adults (*M* = 866 ms, *SD* = 217 ms). However, when processing speed was included as a covariate, this effect became non-significant (*p* = .199). There were no further significant main effects or interactions for RTs for happy vs. neutral faces.

#### Angry vs. neutral faces

**Figure 5.**
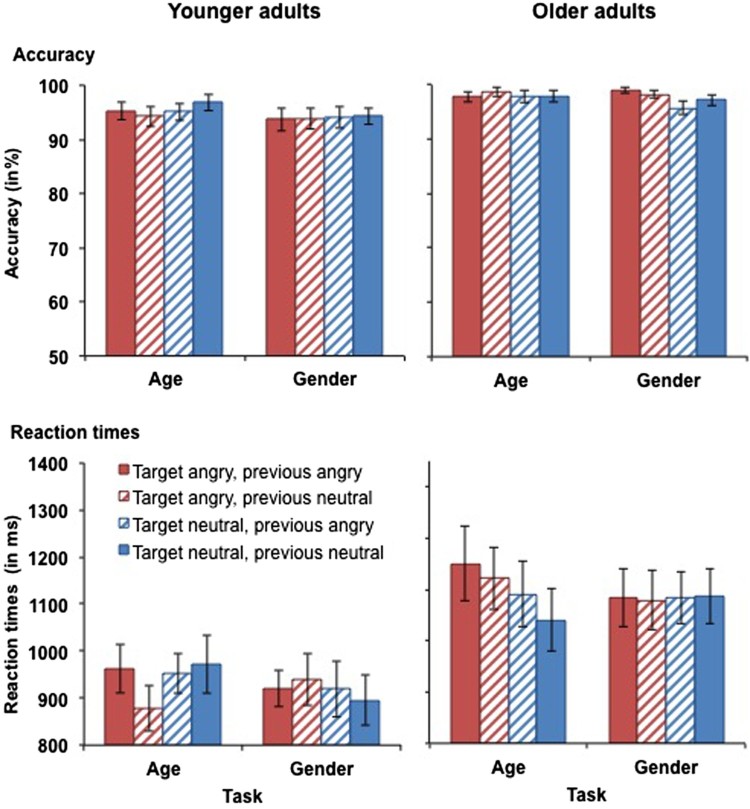
Accuracy (upper panel) and RTs for correct responses (lower panel) in younger (left-hand panel) and older adults (right-hand panel) as a function of target emotion and previous emotion in Experiment 2. Participants switched between the age and the gender tasks with block-wise irrelevant emotion. This figure shows data from the angry vs. neutral task block. Only switch trials are presented. Error bars represent SEM.

***Accuracy.*** Accuracy scores for performance in the angry vs. neutral task block in younger and older adults are presented in Figure 5 (top panels). The analysis yielded a main effect of age, *F*(1, 59) = 6.72, *MSE* = .017, *p* = .012, partial *η^2^* = .10, as older adults were more accurate (*M* = 97.8%, *SD* = 2.5%) than younger adults (*M* = 94.7%, *SD* = 6.2%). No further significant main effects or interactions were observed for accuracy for angry vs. neutral faces.

***Reaction times.*** RTs for performance in the angry vs. neutral task block in younger and older adults are presented in Figure 5 (bottom panels). There was a trend for a task × target emotion × age interaction, *F*(1, 59) = 3.48, *MSE* = 23123, *p* = .067, partial *η^2^* = .06. Separate analyses for the two tasks revealed a trend for an age × target emotion interaction in the age task, *F*(1, 31) = 3.34, *MSE* = 28387, *p* = .073, partial *η^2^* = .06, but not in the gender task (*p* = .439). In the age task, the interaction was driven by older adults’ slower RTs for angry (*M* = 1186 ms, *SD* = 352 ms) rather than neutral targets (*M* = 1116 ms, *SD* = 335 ms), *t*(31) = 2.56, *p* = .016, whereas the difference was non-significant in younger adults (*p* = .016). There was also a main effect of age, *F*(1, 59) = 9.14, *MSE* = 594156, *p* = .004, partial *η^2^* = .13, driven by slower RTs in older (*M* = 1142 ms, *SD* = 307 ms) than in younger adults (*M* = 930 ms, *SD* = 229 ms). However, when processing speed was included as a covariate, the effect became non-significant (*p* = .477). No further significant main effects or interactions were observed for RTs for angry vs. neutral faces.

### Discussion

The aim of Experiment 2 was to assess switching between two non-emotional tasks in the presence of block-wise irrelevant emotion in two age groups. The results revealed that there was no overall effect of block-wise irrelevant emotion on performance in either of the two non-emotional tasks. This can help to interpret findings from Experiment 1, in which both age groups showed a slowdown for emotional relative to neutral items when switching away from an emotional to a non-emotional task. Given that no such effect of emotion was observed when participants had to switch between two non-emotional tasks in Experiment 2, the data suggest that emotion has to be task-relevant for one of the competing tasks in order to affect performance in the non-emotional task. This is in accordance with research showing that consistently irrelevant emotional distractors do not interfere with working memory performance to a greater extent than neutral distractors (Miendlarzewska, Van Elswijk, Cannistraci, & van Ee, 2013; Mullin et al., 2012; Ozawa, Matsuda, & Hiraki, 2014). The present findings also provide strong evidence against the assumption that participants were slower to perform the age task in the presence of emotional rather than neutral faces in the previous experiment due to difficulties to identify the age of an emotional face. No such slowdown for identifying the face's age was observed in Experiment 2 despite the fact that emotional expressions were shown. Although older adults still showed slightly increased RTs for angry relative to neutral targets in the present experiment's age task, this effect was not found to be statistically reliable and was also not observed in the gender task. This finding could indicate that older adults processed negative information even if it was block-wise irrelevant, but the effect does not seem to generalise to all non-emotional tasks. Future studies could examine whether and under which circumstances older adults are affected to a greater extent than younger adults by block-wise irrelevant negative information in task switching.

### General discussion

The present research was conducted to assess the effects of emotion on task switching in younger and older adults and yielded the following main findings: First, when switching between emotional and non-emotional tasks, participants showed slower switch responses for emotional rather than neutral faces in the non-emotional task, with no age-related differences. Second, when switching between two non-emotional tasks, there was no such slowdown in switch responses for emotional compared to neutral faces. Taken together, the results showed that the impairing effect of task-irrelevant emotion on task switching is restricted to cases in which emotion is relevant for one of the competing tasks (i.e. trial-irrelevant emotion) and does not extend to cases in which emotion is consistently irrelevant (i.e. block-irrelevant emotion). This suggests that emotional cues can delay the reconfiguration of a non-emotional task set through the reactivation of the competing emotional task set.

#### Impairing effects of task-irrelevant emotion on task switching

The present study is the first to show that task-irrelevant emotion can contribute to slower switches compared to neutral task-irrelevant material, but only when it is relevant for one of the competing tasks. Importantly, the impairment emerged for the comparison between emotional and neutral items in the non-emotional task, despite the fact that switches to the non-emotional task set were faster than to the emotional task set. This pattern of results might help to explain the inconsistent findings of previous studies, which have exclusively focused on switches between emotional and non-emotional task sets without taking into account the valence of individual items in their analyses (Aboulafia-Brakha et al., 2016; Gul & Khan, 2014; Johnson, 2009; Paulitzki et al., 2008; Reeck & Egner, 2015; Schuch et al., 2012).

Results showing that switching from an emotional to a non-emotional task comes at a greater cost than vice versa (Johnson, 2009; Paulitzki et al., 2008) were usually interpreted as evidence that disengaging from an emotional rather than non-emotional task is more difficult and/or that engaging with an emotional rather than non-emotional task is less difficult. However, switches from the emotional to the non-emotional task were not slower than vice versa in the present study, and thus, the data provide no support for this interpretation. In contrast, there is also evidence that costs are greater for switches to an emotional from a non-emotional task than vice versa (Reeck & Egner, 2015), which was interpreted as evidence for the inhibition of the more dominant emotional task set to facilitate performance in a less dominant non-emotional task. As no neutral items were used in the study by Reeck and Egner (2015), their design did not allow assessing whether emotion was indeed inhibited by comparing switch responses to neutral and emotional items in the non-emotional task. It should be noted that in the present study, switch costs were greater when participants had to switch from a non-emotional to an emotional task, which is similar to the results reported by Reeck and Egner (2015). However, the data do not support an interpretation based on inhibition of the emotional task set. In the present research, emotional faces slowed down switches from an emotional to a non-emotional task, which suggests that there was no (or at least no successful) emotion inhibition. Overall, it appears that without distinguishing responses to neutral and emotional items, the interpretation of the effects of emotion on task switching can only be inconsistent or incomplete at best.

#### The effects of emotion on task set reconfiguration vs. task set interference

Taking into account the stimulus valence in a task-switching paradigm can help disentangling the effects of emotion on sub-mechanisms involved in task switching. The literature suggests that both the reconfiguration of the new task set and the interference of the previous task set are relevant for task switching. Whereas the reconfiguration account of task switching could explain effects of target emotion on switching, the interference account of task switching is more suitable to explain effects of previous emotion on switching. Experiment 1 revealed that the emotion participants switched to affected their performance, whereas the emotion they switched away from did not. Although this finding does not rule out that interference played a role in task switching in Experiment 1, the pattern of results suggest that it was not affected by emotion. This is not compatible with suggestions that emotional processing is suppressed when participants switch between emotional and non-emotional tasks (Reeck & Egner, 2015).

Instead, the results highlight effects of emotion on task set reconfiguration as it appears that the reconfiguration of a non-emotional task set was less efficient in the presence of trial-irrelevant emotion. As the slowdown in task switching was observed for emotional relative to neutral items, it is likely that emotional items triggered the competing emotional task set, whereas neutral items did not or less so. This reactivation of the competing emotional task set in turn interfered with the reconfiguration of the non-emotional task set. It also appears that both happy and angry faces triggered reactivation, pointing at an effect of arousal rather than valence. Overall, these results are in accordance with the suggestion that exogenous factors play an important role in task switching through task cueing (Kiesel et al., 2010; Rogers & Monsell, 1995; Rubin & Koch, 2006). As reviewed by Kiesel et al. (2010), stimulus-based task activation can happen at the level of the response (i.e. items are processed according to the stimulus-response mapping of the alternative task) or at the level of the abstract task set. Given that responses to each of the two tasks were assigned to different hands in the present research (i.e. different stimulus-response mappings), it appears that reactivation of the emotional task set happened at the level of the abstract task set and not at the response level.

As mentioned above, the reactivation of the competing task set not only affected switch trials from the emotional to the non-emotional task but also repeat trials. It is not clear whether this was a short-term “spill-over” from switch trials or whether it was a longer-lasting reactivation effect. In the present study, only one repeat trial per task was included and thus, it is difficult to evaluate the effect's longevity. Future studies could explore whether the reactivation of the competing task set diminishes with an increasing number of repeat trials or not (e.g. when participants switch every four rather than every two trials). The former would speak in favour of a short-term effect, whereas the latter would suggest that there is sustained reactivation of the competing task set. Furthermore, future studies could investigate whether higher flexibility of emotional relative to non-emotional task sets as reported by Schuch et al. (2012) is associated with emotional task sets’ greater susceptibility to reactivation that were observed in the present study. Also, stimuli were presented repeatedly in our study, which could have allowed for rapid stimulus-response learning (e.g. Schnyer et al., 2007). As the association of a stimulus with a particular response can facilitate and impair subsequent responses and item presentation was random in our study, it is unlikely that rapid stimulus-response learning can account for the impairing effects of task-irrelevant emotion on task switching. However, future studies could assess the role of stimulus-response learning in task set reconfiguration.

#### No age-related differences in the effects of task-irrelevant emotion on task switching

This research was the first to examine the effects of emotion on task switching in ageing and no age-related differences were observed. Contrary to the hypotheses, happy faces did not facilitate engagement with or the disengagement from a task in older relative to younger adults. Moreover, there was no evidence of age-related impairments in performance, which is in line with research showing that local task switching is largely unaffected in ageing (for a meta-analysis, see Wasylyshyn et al., 2011). The present research was not designed to distinguish between different theoretical accounts that have been suggested to explain how emotion-cognition interactions change with age. However, it is possible to speculate on the suitability of different accounts based on the present findings.

Older adults’ very high accuracy rates in the two experiments suggest that they were not functioning at the limit of their cognitive capacity and that additional cognitive resources were likely available. Despite the availability of cognitive resources, emotion did not affect older and younger adults differently. This does not directly support the SST (Carstensen, 1993), according to which older adults use available cognitive resources to focus on emotional and particularly positive information in order to enhance wellbeing. The results are only reconcilable with the SST when considering that specific task goals may supplant chronically active emotion regulation goals in older adults (for a review, see Reed & Carstensen, 2012). In the present study, specific instructions were used to direct the participants’ attention to particular item features (i.e. emotion, age or gender of faces), which might have hindered the processing of emotional stimuli in a motivation-based way. It should be noted, however, that studies with similar restrictive task instructions in the domain of working memory have observed age-related differences in emotion-cognition interactions and interpreted these within the framework of SST (e.g. Borg, Leroy, Favre, Laurent, & Thomas-Antérion, 2011; Truong & Yang, 2014). Thus, it is difficult to assess the validity of the theory if the role of task instructions for age-related emotion biases is not consistently considered as a relevant factor. In contrast, the data are fully compatible with the dynamic integration theory (Labouvie-Vief, 2003, 2009; Labouvie-Vief & González, 2004), which suggests that emotional biases in ageing reflect a compensatory strategy as they help to buffer the effects of cognitive decline. As the task was not too difficult for older adults, it is likely that no compensation for insufficient cognitive resources through biased processing of emotional material was needed. Future studies should explore this further by testing whether age-related changes in the effects of emotion on task switching emerge with increased task difficulty, which would provide support for a decline-based account.

#### Conclusion

To conclude, the present study was the first to investigate the effects of emotion on task switching in younger and older adults. The study revealed that task-irrelevant emotion impaired switching in both age groups, but only when emotion was relevant for one of the competing tasks. The results suggest that the impairing effect of task-irrelevant emotion in a non-emotional task was due to the reactivation of the competing emotional task. They also suggest that local task switching and the ability to shield task-switching performance from block-wise irrelevant emotion are preserved in ageing.

## Supplementary Material

Supplementary_Material

## References

[CIT0001] Aboulafia-Brakha T., Manuel A. L., & Ptak R. (2016). Prefrontal transcranial direct current stimulation facilitates affective flexibility. *Neuropsychologia*, 86, 13–18. doi: 10.1016/j.neuropsychologia.2016.03.030 27039163

[CIT0002] Allport D. A., Styles E. A., & Hsieh S. (1994). Shifting intentional set: Exploring the dynamic control of tasks. In Umiltà C., & Moscovitch M. (Eds.), *Attention and performance 15: Conscious and nonconscious information processing* (pp. 421–452). Cambridge, MA: MIT Press.

[CIT0003] Alwin D. F., & McCammon R. J. (2001). Aging, cohorts, and verbal ability. *The Journals of Gerontology: Series B*, 56(3), S151–S161. doi: 10.1093/geronb/56.3.S151 11316840

[CIT0004] Babcock R. L., & Salthouse T. A. (1990). Effects of increased processing demands on age differences in working memory. *Psychology and Aging*, 5, 421–428. doi: 10.1037/0882-7974.5.3.421 2242246

[CIT0005] Bach M. (1996). The Freiburg visual acuity test – Automatic measurement of visual acuity. *Optometry and Vision Science*, 73, 49–53. doi:1040-5488/96/7301-0049$03.00/0 doi: 10.1097/00006324-199601000-00008 8867682

[CIT0006] Beck A. T., Steer R. A., & Carbin M. G. (1988). Psychometric properties of the Beck Depression Inventory: Twenty-five years of evaluation. *Clinical Psychology Review*, 8, 77–100. doi: 10.1016/0272-7358(88)90050-5

[CIT0007] Berger N., Richards A., & Davelaar E. J. (2017). When emotions matter: Focusing on emotion improves working memory updating in older adults. *Frontiers in Psychology*, 8, 1565. doi: 10.3389/fpsyg.2017.01565 28966602 PMC5605649

[CIT0008] Blanchard-Fields F. (2007). Everyday problem solving and emotion: An adult developmental perspective. *Current Directions in Psychological Science*, 16, 26–31. doi: 10.1111/j.1467-8721.2007.00469.x

[CIT0009] Blanchard-Fields F., Mienaltowski A., & Seay R. B. (2007). Age differences in everyday problem-solving effectiveness: Older adults select more effective strategies for interpersonal problems. *The Journals of Gerontology Series B: Psychological Sciences and Social Sciences*, 62, P61–P64. doi: 10.1093/geronb/62.1.P61 17284559

[CIT0010] Borg C., Leroy N., Favre E., Laurent B., & Thomas-Antérion C. (2011). How emotional pictures influence visuospatial binding in short-term memory in ageing and Alzheimer’s disease? *Brain and Cognition*, 76, 20–25. doi: 10.1016/j.bandc.2011.03.008 21481999

[CIT0011] Bowles R. P., Grimm K. J., & McArdle J. J. (2005). A structural factor analysis of vocabulary knowledge and relations to age. *The Journals of Gerontology Series B: Psychological Sciences and Social Sciences*, 60(5), P234–P241. doi: 10.1093/geronb/60.5.P234 16131617

[CIT0012] Braver T. S., & West R. (2008). Working memory, executive control, and aging. In Craik F. I. M., & Salthouse T. A. (Eds.), *The handbook of aging and cognition* (3rd ed., pp. 311–372). New York, NY: Psychology Press.

[CIT0013] Breiter H. C., Etcoff N. L., Whalen P. J., Kennedy W. A., Rauch S. L., Buckner R. L., … Rosen B. R. (1996). Response and habituation of the human amygdala during visual processing of facial expression. *Neuron*, 17, 875–887. doi: 10.1016/S0896-6273(00)80219-6 8938120

[CIT0014] Carstensen L. L. (1993). Motivation for social contact across the life span: A theory of socioemotional selectivity. In Jacobs J. E. (Ed.), *Nebraska symposium on motivation: Developmental perspectives on motivation* (Vol. 40, pp. 209–254). Lincoln, NE: University of Nebraska Press. 1340521

[CIT0015] Carstensen L. L., Pasupathi M., Mayr U., & Nesselroade J. R. (2000). Emotional experience in everyday life across the adult life span. *Journal of Personality and Social Psychology*, 79, 644–655. doi: 10.1037/0022-3514.79.4.644 11045744

[CIT0016] de Vries M., & Geurts H. M. (2012). Cognitive flexibility in ASD; task switching with emotional faces. *Journal of Autism and Developmental Disorders*, 42, 2558–2568. doi: 10.1007/s10803-012-1512-1 22456815 PMC3490074

[CIT0017] Ebner N. C., Riediger M., & Lindenberger U. (2010). FACES—A database of facial expressions in young, middle-aged, and older women and men: Development and validation. *Behavior Research Methods*, 42, 351–362. doi: 10.3758/BRM.42.1.351 20160315

[CIT0018] Folstein M. F., Folstein S. E., & McHugh P. R. (1975). Mini mental state’. A practical method for grading the cognitive state of patients for the clinician. *Journal of Psychiatric Research*, 12, 189–198. doi: 10.1016/0022-3956(75)90026-6 1202204

[CIT0019] Ganel T. (2015). Smiling makes you look older. *Psychonomic Bulletin & Review*, 22, 1671–1677. doi: 10.3758/s13423-015-0822-7 25855200

[CIT0020] Gross J. J., Carstensen L. L., Pasupathi M., Tsai J., Götestam Skorpen C., & Hsu A. Y. C. (1997). Emotion and aging: Experience, expression, and control. *Psychology and Aging*, 12, 590–599. doi: 10.1037/0882-7974.12.4.590 9416628

[CIT0021] Gul A., & Khan K. (2014). Emotion regulation strategies can predict task-switching abilities in euthymic bipolar patients. *Frontiers in Human Neuroscience*, 8, 847. doi: 10.3389/fnhum.2014.00847 25386129 PMC4209808

[CIT0022] Johnson D. R. (2009). Emotional attention set-shifting and its relationship to anxiety and emotion regulation. *Emotion*, 9, 681–690. doi: 10.1037/a0017095 19803590

[CIT0023] Kiesel A., Steinhauser M., Wendt M., Falkenstein M., Jost K., Philipp A. M., & Koch I. (2010). Control and interference in task switching—A review. *Psychological Bulletin*, 136, 849–874. doi: 10.1037/a0019842 20804238

[CIT0024] Kray J., Li K. Z. H., & Lindenberger U. (2002). Age-related changes in task-switching components: The role of task uncertainty. *Brain and Cognition*, 49(3), 363–381. doi: 10.1006/brcg.2001.1505 12139959

[CIT0025] Labouvie-Vief G. (2003). Dynamic integration: Affect, cognition, and the self in adulthood. *Current Directions in Psychological Science*, 12, 201–206. doi: 10.1046/j.0963-7214.2003.01262.x

[CIT0026] Labouvie-Vief G. (2009). Dynamic integration theory: Emotion, cognition, and equilibrium in later life. In Bengston V. L., Gans D., Pulney N. M., & Silverstein M. (Eds.), *Handbook of theories of aging* (2nd ed., pp. 277–293). New York, NY: Springer Publishing Co.

[CIT0027] Labouvie-Vief G., & González M. M. (2004). Dynamic integration: Affect optimization and differentiation in development. In *Motivation, emotion, and cognition: Integrative perspectives on intellectual functioning and development* (pp. 237–272). Mahwah, NJ: Lawrence Erlbaum Associates Publishers.

[CIT0028] Larcom M. J., & Isaacowitz D. M. (2009). Rapid emotion regulation after mood induction: Age and individual differences. *The Journals of Gerontology Series B: Psychological Sciences and Social Sciences*, 64B(6), 733–741. doi: 10.1093/geronb/gbp077 PMC2763016 19808810

[CIT0029] MacPherson S. E., Phillips L. H., & Della Sala S. (2002). Age, executive function and social decision making: A dorsolateral prefrontal theory of cognitive aging. *Psychology and Aging*, 17, 598–609. doi: 10.1037/0882-7974.17.4.598 12507357

[CIT0030] Mayr U., & Kliegl R. (2000). Task-set switching and long-term memory retrieval. *Journal of Experimental Psychology: Learning, Memory, and Cognition*, 26, 1124–1140. doi: 10.1037/0278-7393.26.5.1124 11009248

[CIT0031] Meiran N., Chorev Z., & Sapir A. (2000). Component processes in task switching. *Cognitive Psychology*, 41(3), 211–253. doi: 10.1006/cogp.2000.0736 11032657

[CIT0032] Miendlarzewska E. A., Van Elswijk G., Cannistraci C. V., & van Ee R. (2013). Working memory load attenuates emotional enhancement in recognition memory. *Frontiers in Psychology*, 4, 1–10. doi: 10.3389/fpsyg.2013.00112 23515565 PMC3600573

[CIT0033] Miyake A., & Friedman N. P. (2012). The nature and organization of individual differences in executive functions four general conclusions. *Current Directions in Psychological Science*, 21, 8–14. doi: 10.1177/0963721411429458 22773897 PMC3388901

[CIT0034] Miyake A., Friedman N. P., Emerson M. J., Witzki A. H., Howerter A., & Wager T. D. (2000). The unity and diversity of executive functions and their contributions to complex “frontal lobe” tasks: A latent variable analysis. *Cognitive Psychology*, 41, 49–100. doi: 10.1006/cogp.1999.0734 10945922

[CIT0035] Monsell S. (2003). Task switching. *Trends in Cognitive Sciences*, 7(3), 134–140. doi: 10.1016/S1364-6613(03)00028-7 12639695

[CIT0036] Mullin B. C., Perlman S. B., Versace A., de Almeida J. R. C., LaBarbara E. J., Klein C., … Phillips M. L. (2012). An fMRI study of attentional control in the context of emotional distracters in euthymic adults with bipolar disorder. *Psychiatry Research: Neuroimaging*, 201, 196–205. doi: 10.1016/j.pscychresns.2011.09.002 PMC3361638 22510433

[CIT0037] Nelson H. E., & Willison J. (1991). *National Adult Reading Test (NART): Test manual*. Windsor: Nfer-Nelson.

[CIT0038] Ozawa S., Matsuda G., & Hiraki K. (2014). Negative emotion modulates prefrontal cortex activity during a working memory task: A NIRS study. *Frontiers in Human Neuroscience*, 8, 46. doi: 10.3389/fnhum.2014.00046 24574991 PMC3918646

[CIT0039] Paulitzki J. R., Risko E. F., Oakman J. M., & Stolz J. A. (2008). Doing the unpleasant: How the emotional nature of a threat-relevant task affects task-switching. *Personality and Individual Differences*, 45(5), 350–355. doi: 10.1016/j.paid.2008.05.003

[CIT0040] Pessoa L. (2009). How do emotion and motivation direct executive control? *Trends in Cognitive Sciences*, 13(4), 160–166. doi: 10.1016/j.tics.2009.01.006 19285913 PMC2773442

[CIT0041] Pessoa L. (2015). The cognitive-emotional amalgam. *Behavioral and Brain Sciences*, 38, 1–66. doi:10.1017/S0140525X14000120 doi: 10.1017/S0140525X14000120 26815655

[CIT0042] Pessoa L. (2017). Cognitive control and emotional processing. In Egner T. (Ed.), *The Wiley handbook of cognitive control* (pp. 394–409). Chichster, West Sussex: John Wiley & Sons, Ltd.

[CIT0043] Phan K. L., Liberzon I., Welsh R. C., Britton J. C., & Taylor S. F. (2003). Habituation of rostral anterior cingulate cortex to repeated emotionally salient pictures. *Neuropsychopharmacology*, 28(7), 1344–1350. doi: 10.1038/sj.npp.1300186 12784119

[CIT0044] Phelps E. A., & LeDoux J. E. (2005). Contributions of the amygdala to emotion processing: From animal models to human behavior. *Neuron*, 48(2), 175–187. doi: 10.1016/j.neuron.2005.09.025 16242399

[CIT0045] Phelps E. A., Ling S., & Carrasco M. (2006). Emotion facilitates perception and potentiates the perceptual benefits of attention. *Psychological Science*, 17(4), 292–299. doi: 10.1111/j.1467-9280.2006.01701.x 16623685 PMC1555625

[CIT0046] Piguet C., Cojan Y., Sterpenich V., Desseilles M., Bertschy G., & Vuilleumier P. (2016). Alterations in neural systems mediating cognitive flexibility and inhibition in mood disorders. *Human Brain Mapping*, 37, 1335–1348. doi: 10.1002/hbm.23104 26787138 PMC6867498

[CIT0047] Piguet C., Sterpenich V., Desseilles M., Cojan Y., Bertschy G., & Vuilleumier P. (2013). Neural substrates of cognitive switching and inhibition in a face processing task. *NeuroImage*, 82, 489–499. doi: 10.1016/j.neuroimage.2013.06.015 23774397

[CIT0048] Reeck C., & Egner T. (2015). Emotional task management: Neural correlates of switching between affective and non-affective task-sets. *Social Cognitive and Affective Neuroscience*, 10(8), 1045–1053. doi: 10.1093/scan/nsu153 25552571 PMC4526474

[CIT0049] Reed A. E., & Carstensen L. L. (2012). The theory behind the age-related positivity effect. *Frontiers in Psychology*, 3, 1–9. doi: 10.3389/fpsyg.2012.00339 23060825 PMC3459016

[CIT0050] Reimers S., & Maylor E. A. (2005). Task switching across the life span: Effects of age on general and specific switch costs. *Developmental Psychology*, 41, 661–671. doi: 10.1037/0012-1649.41.4.661 16060812

[CIT0051] Reuter-Lorenz P. A., & Sylvester C.-Y. C. (2005). The cognitive neuroscience of working memory and aging. In Cabeza R., Nyberg L., & Park D. (Eds.), *Cognitive neuroscience of aging: Linking cognitive and cerebral aging* (pp. 186–217). New York, NY: Oxford University Press.

[CIT0052] Rogers R. D., & Monsell S. (1995). Costs of a predictible switch between simple cognitive tasks. *Journal of Experimental Psychology: General*, 124, 207–231. doi: 10.1037/0096-3445.124.2.207

[CIT0053] Rubin O., & Koch I. (2006). Exogenous influences on task set activation in task switching. *Quarterly Journal of Experimental Psychology*, 59, 1033–1046. doi: 10.1080/02724980543000105 16885142

[CIT0054] Salthouse T. A. (1990). Working memory as a processing resource in cognitive aging. *Developmental Review*, 10, 101–124. doi: 10.1016/0273-2297(90)90006-P

[CIT0055] Salthouse T. A. (1991). *Theoretical perspectives on cognitive aging*. Hillsdale, NJ: Lawrence Erlbaum Associates, Inc.

[CIT0056] Salthouse T. A. (1996). The processing-speed theory of adult age differences in cognition. *Psychological Review*, 103, 403–428. doi: 10.1037/0033-295X.103.3.403 8759042

[CIT0057] Salthouse T. A. (2000). Aging and measures of processing speed. *Biological Psychology*, 54, 35–54. doi: 10.1016/S0301-0511(00)00052-1 11035219

[CIT0058] Scheibe S., & Blanchard-Fields F. (2009). Effects of regulating emotions on cognitive performance: What is costly for young adults is not so costly for older adults. *Psychology and Aging*, 24, 217–223. doi: 10.1037/a0013807 19290754 PMC2658623

[CIT0059] Scheibe S., & Carstensen L. L. (2010). Emotional aging: Recent findings and future trends. *The Journals of Gerontology Series B: Psychological Sciences and Social Sciences*, 65B(2), 135–144. doi: 10.1093/geronb/gbp132 PMC2821944 20054013

[CIT0060] Schneider W., Eschman A., & Zuccolotto A. (2002). *E-Prime: User’s guide*. Pittsburgh: Psychology Software Tools Inc.

[CIT0061] Schnyer D. M., Dobbins I. G., Nicholls L., Davis S., Verfaellie M., & Schacter D. L. (2007). Item to decision mapping in rapid response learning. *Memory & Cognition*, 35(6), 1472–1482. doi: 10.3758/BF03193617 17948070 PMC2034352

[CIT0062] Schuch S., Werheid K., & Koch I. (2012). Flexible and inflexible task sets: Asymmetric interference when switching between emotional expression, sex, and age classification of perceived faces. *Quarterly Journal of Experimental Psychology*, 65(5), 994–1005. doi: 10.1080/17470218.2011.638721 22339339

[CIT0063] Spielberger C. D., Gorsuch R. L., Lushene R., Vagg P. R., & Jacobs G. A. (1983). *Manual for the state-trait anxiety inventory*. Palo Alto, CA: Consulting Psychologists Press.

[CIT0064] Truong L., & Yang L. (2014). Friend or foe? Decoding the facilitative and disruptive effects of emotion on working memory in younger and older adults. *Frontiers in Psychology*, 5, 94. doi: 10.3389/fpsyg.2014.00094 24624097 PMC3933777

[CIT0065] Verhaeghen P., & Cerella J. (2002). Aging, executive control, and attention: A review of meta-analyses. *Neuroscience and Biobehavioral Reviews*, 26, 849–857. doi: 10.1016/S0149-7634(02)00071-4 12470697

[CIT0066] Voelkle M. C., Ebner N. C., Lindenberger U., & Riediger M. (2012). Let me guess how old you are: Effects of age, gender, and facial expression on perceptions of age. *Psychology and Aging*, 27, 265–277. doi: 10.1037/a0025065 21895379

[CIT0067] Wasylyshyn C., Verhaeghen P., & Sliwinski M. J. (2011). Aging and task switching: A meta-analysis. *Psychology and Aging*, 26(1), 15–20. doi: 10.1037/a0020912 21261411 PMC4374429

[CIT0068] Wechsler D. (1955). *Der Hamburg Wechsler Intelligenztest für Erwachsene (HAWIE)*. Bern: Huber.

[CIT0069] Wylie G., & Allport A. (2000). Task switching and the measurement of “switch costs”. *Psychological Research*, 63(3–4), 212–233. doi: 10.1007/s004269900003 11004877

[CIT0070] Zald D. H. (2003). The human amygdala and the emotional evaluation of sensory stimuli. *Brain Research Reviews*, 41(1), 88–123. doi: 10.1016/S0165-0173(02)00248-5 12505650

[CIT0071] Zelazo P. D., Craik F. I. M., & Booth L. (2004). Executive function across the life span. *Acta Psychologica*, 115(2–3), 167–183. doi: 10.1016/j.actpsy.2003.12.005 14962399

