# Supplementary material for: Delayed reconfiguration of a non-emotional task set through reactivation of an emotional task set in task switching: an ageing study
Source: Cogn Emot. 2019 Jan 17;33(7):1370–86. doi: 10.1080/02699931.2019.1567462 (PMC6816485; doi:10.1080/02699931.2019.1567462)
Supplement: Supplementary_Material [file PCEM_A_1567462_SM4498.doc]

# Supplemental Material

## Results for repeat trials, Experiment 1

#### **Statistical analysis**

Statistical analysis of the data from repeat trials was conducted with SPSS 22 (IBM Corp., Armonk, NY). Accuracy and RTs for repeat trials were analysed separately for the happy vs. neutral and the angry vs. neutral blocks. Data were analysed in the same way as for switch trials by a 2 × 2 × 2 × 2 mixed factors ANOVA including the within-subjects factors task (age task vs. emotion task), target emotion (happy/angry vs. neutral) and previous emotion (happy/angry vs. neutral) and the between-subjects factor of age (younger vs. older). Post-hoc t*-*tests with a Bonferroni adjustment to the 5% alpha level were performed to follow up significant interactions.

#### **Happy vs. neutral faces**

***Accuracy.*** The four-way omnibus ANOVA on repeat trials yielded a main effect of age, *F*(1, 59) = 15.05, *MSE* = .003, *p* < .001, partial *η^2^* = .20, as older adults were more accurate (*M* = 99.2 %, *SD* = 1.2 %) than younger adults (*M* = 97.2 %, *SD* = 2.7 %). No further main effects or interactions were observed for accuracy for happy vs. neutral faces.

***Reaction times.*** The four-way omnibus ANOVA on repeat trials yielded a main effect of task, *F*(1, 59) = 7.56, *MSE* = 32260, *p* = .008, partial *η^2^* = .11, with overall slower RTs in the emotion task (*M* = 822 ms, *SD* = 223 ms) compared to the age task (*M* = 777 ms, *SD* = 224 ms). This main effect was qualified by a task × target emotion interaction, *F*(1, 59) = 7.83, *MSE* = 12716, *p* = .007, partial *η^2^* = .11. Follow-up t-tests revealed that in the age task, RTs were slower for happy targets (*M* = 798 ms, *SD* = 243 ms) than for neutral targets (*M* = 757 ms, *SD* = 216 ms), *t*(60) = 3.04, *p* = .004. In the emotion task, there was no significant difference between RTs for neutral or happy targets (*p* = .289). There was also a target emotion × previous emotion interaction, *F*(1, 59) = 10.57, *MSE* = 18416, *p* = .002, partial *η^2^* = .15. Follow-up t-tests revealed that RTs for neutral targets were faster when the previous emotion was also neutral (*M* = 735 ms, *SD* = 216 ms) rather than happy (*M* = 778 ms, *SD* = 243 ms), *t*(60) = 2.14, *p* = .036. Similarly, RTs for happy targets were faster when the previous emotion was also happy (*M* = 767 ms, *SD* = 226 ms) rather than neutral (*M* = 827 ms, *SD* = 289 ms), *t*(60) = 2.45, *p* = .017. This is evidence for a response repetition effect for repeat trials. Finally, there was also a main effect of age, *F*(1, 59) = 17.12, *MSE* = 290092, *p* < .001, partial *η^2^* = .26, as older adults were overall slower (*M* = 896 ms, *SD* = 217 ms) than younger adults (*M* = 694 ms, *SD* = 167 ms). No further main effects or interactions were observed for reaction times to happy vs. neutral faces.

#### **Angry vs. neutral faces**

***Accuracy.*** The four-way omnibus ANOVA on repeat trials yielded a main effect of task, *F*(1, 59) = 7.07, *MSE* = .010, *p* = .010, partial *η^2^* = .11, as accuracy scores were higher in the age task (*M* = 97.5 %, *SD* = 3.6 %) than in the emotion task (*M* = 95.8 %, *SD* = 4.5 %). There was also a main effect of target emotion, *F*(1, 59) = 10.37, *MSE* = .004, *p* = .002, partial *η^2^* = .15, with lower accuracy scores for angry targets (*M* = 95.7 %, *SD* = 4.6 %) than for neutral targets (*M* = 97.6 %, *SD* = 3.1 %). This main effect was qualified by a task × target emotion interaction, *F*(1, 59) = 6.63, *MSE* = .005, *p* = .013, partial *η^2^* = .10. Follow-up t-tests revealed that in the emotion task, accuracy was lower for angry targets (*M* = 94.0 %, *SD* = 7.6 %) than for neutral targets (*M* = 97.5 %, *SD* = 4.0 %), *t*(60) = 3.42, *p* = .001, whereas accuracy for neutral and angry targets did not differ in the age task (*p* = .713). There was also a task × previous emotion interaction, *F*(1, 59) = 7.47, *MSE* = .004, *p* = .008, partial *η^2^* = .11, which was qualified by a marginally significant task × previous emotion × age interaction, *F*(1, 57) = 3.94, *MSE* = .004, *p* = .052, partial *η^2^* = .07. Separate analyses for the emotion and age tasks were conducted to follow up on this interaction. In the emotion task, there was a significant previous emotion × age interaction, *F*(1, 57) = 4.70, *MSE* = .005, *p* = .034, partial *η^2^* = .08, whereas no such effect was found in the age task (*p* = .614). In the emotion task, younger adults were more accurate if the previous emotion was angry (*M* = 96.4 %, *SD* = 5.8 %) rather than neutral (*M* = 92.2 %, *SD* = 6.8 %), *t*(28) = 3.19, *p* = .003, whereas previous emotion did not play such a role for older adults (*p* = .312). There was a also a main effect of age, *F*(1, 59) = 17.52, *MSE* = .006, *p* < .001, partial *η^2^* = .23, as older adults were more accurate (*M* = 98.1 %, *SD* = 2.2 %) than younger adults (*M* = 95.1 %, *SD* = 3.4 %). No further main effects or interaction were observed for accuracy scores for angry vs. neutral faces.

***Reaction times.*** The four-way omnibus ANOVA on repeat trials yielded a main effect of task, *F*(1, 59) = 24.23, *MSE* = 49550, *p* < .001, partial *η^2^* = .29, as participants were slower in the emotion task (*M* = 880 ms, *SD* = 225 ms) than in the age task (*M* = 781 ms, *SD* = 243 ms). There was also a main effect of target emotion, *F*(1, 59) = 11.03, *MSE* = 24199, *p* = .002, partial *η^2^* = .16, with slower RTs for angry targets (*M* = 854 ms, *SD* = 239 ms) relative to neutral targets (*M* = 806 ms, *SD* = 216 ms). Although the task × target emotion interaction was not significant (*p* = .217), separate analyses for the age task and for the emotion task were conducted to assess whether the effect of emotion differed in size in the two task. This was done as in the switch trials for happy/neutral and angry/neutral faces and in the repeat trials for happy/neutral faces, emotion affected performance on the non-emotional age task. The separate analyses revealed that in the age task, RTs were slower for angry targets (*M* = 814 ms, *SD* = 261 ms) than for neutral targets (*M* = 748 ms, *SD* = 238 ms), *t*(60) = 4.57, *p* < .001. In the emotion task, the difference in RTs for neutral targets (*M* = 864 ms, *SD* = 220 ms) and angry targets (*M* = 895 ms, *SD* = 268 ms) was not significant (p = .216).

There was also a target emotion × previous emotion interaction, *F*(1, 59) = 25.98, *MSE* = 16593, *p* < .001, partial *η^2^* = .31, which was qualified by a task × target emotion × previous emotion interaction, *F*(1, 59) = 5.56, *MSE* = 17278, *p* = .022, partial *η^2^* = .09. Separate analyses for the age and emotion tasks were conducted to follow up on this interaction. The target emotion × previous emotion interaction was significant in the age task, *F*(1, 59) = 5.00, *MSE* = 12013, *p* = .029, partial *η^2^* = .08. Follow-up t-tests showed that in the age task, RTs to angry faces were faster if the previous emotion was angry (*M* = 785 ms, *SD* = 262 ms) rather than neutral (*M* = 842 ms, *SD* = 286 ms), *t*(60) = 2.61, *p* = .011, whereas previous emotion did not play a role for responses to neutral targets (*p* = .011). The target emotion × previous emotion interaction was also significant in the age task and the interaction was more pronounced, *F*(1, 59) = 21.37, *MSE* = 21857, *p* < .001, partial *η^2^* = .27. Follow-up t-tests showed that in the emotion task, RTs to angry faces were faster if the previous emotion was angry (*M* = 852 ms, *SD* = 275 ms) rather than neutral (*M* = 939 ms, *SD* = 308 ms), *t*(60) = 2.94, *p* = .005. Similarly, RTs to neutral faces were faster if the previous emotion was neutral (*M* = 852 ms, *SD* = 275 ms) rather than angry (*M* = 939 ms, *SD* = 308 ms), *t*(60) = 4.13, *p* < .001. Again, this pattern of results is evidence for a response repetition effect for repeat trials. Lastly, there was also a main effect of age, *F*(1, 59) = 17.03, *MSE* = 308142, *p* < .001, partial *η^2^* = .22, as older adults were overall slower (*M* = 929 ms, *SD* = 246 ms) than younger adults (*M* = 721 ms, *SD* = 119 ms). No further main effects or interactions were observed for reaction times for angry vs. neutral faces.

## Results for repeat trials, Experiment 2

#### **Statistical analysis**

Statistical analysis of the data from repeat trials was conducted with SPSS 22 (IBM Corp., Armonk, NY). Accuracy and RTs for repeat trials were analysed separately for the happy vs. neutral and the angry vs. neutral blocks. Data were analysed in the same way as for switch trials by a 2 × 2 × 2 × 2 mixed factors ANOVA including the within-subjects factors task (age task vs. gender task), target emotion (happy/angry vs. neutral) and previous emotion (happy/angry vs. neutral) as well as the between-subjects factor of age (younger vs. older). Post-hoc t*-*tests with a Bonferroni adjustment to the 5% alpha level were performed to follow up significant interactions.

#### **Happy vs. neutral faces**

***Accuracy.*** The analysis of accuracy for repeat trials yielded a significant task × target emotion × previous emotion interaction, *F*(1, 59) = 8.61, *MSE* = .003, *p* = .005, partial *η^2^* = .13. Separate analyses for the age and the gender task were conducted to follow up on this interaction. In the age task, the target emotion × previous emotion interaction was significant, *F*(1, 59) = 6.36, *MSE* = .002, *p* = .014, partial *η^2^* = .10, whereas it missed significance in the gender task (*p* = .076). Follow-up t-test revealed that in the age task, participants responded more accurately to neutral targets if the previous emotion was happy (*M* = 98.5 %, *SD* = 3.8 %) rather than neutral (*M* = 96.2 %, *SD* = 7.3 %), *t*(28) = 2.31, *p* = .024. In contrast, previous emotion did not play a similar role in accuracy for happy target faces (*p* = .477). There was also a main effect of age, *F*(1, 59) = 8.96, *MSE* = .004, *p* = .004, as older adults were more accurate (*M* = 98.9 %, *SD* = 1.3 %) than younger adults (*M* = 96.0 %, *SD* = 2.9 %). No further main effects or interactions were observed for accuracy scores for happy vs. neutral faces.

***Reaction times.*** The four-way omnibus ANOVA on repeat trials yielded a significant target emotion × age interaction, *F*(1, 59) = 4.22, *MSE* = 15196, *p* = .044, partial *η^2^* = .07. Follow-up t-test revealed that younger adults responded marginally slower when the target emotion was happy (*M* = 674 ms, *SD* = 149 ms) rather than neutral (*M* = 641 ms, *SD* = 131 ms), *t*(28) = 2.00, *p* = .055. In contrast, there was no significant difference in RTs for neutral or happy targets in older adults (*p* = .472). There was a main effect of age, *F*(1, 59) = 14.48, *MSE* = 237440, *p* < .001, partial *η^2^* = .20, driven by slower RTs in older (*M* = 825 ms, *SD* = 201 ms) than in younger adults (*M* = 657 ms, *SD* = 133 ms). However, when processing speed was included as a covariate, this effect became non- significant (*p* = .216). No further main effects or interactions were observed for reaction times to happy vs. neutral faces.

#### **Angry vs. neutral faces**

***Accuracy.*** The analysis of accuracy for repeat trials yielded a task × target emotion interaction, *F*(1, 59) = 7.02, *MSE* = .003, *p* = .010, partial *η^2^* = .11. Follow-up t-test revealed that in the gender task, participants responded more accurately to angry targets (*M* = 98.3 %, *SD* = 3.6 %) than to neutral targets (*M* = 96.6 %, *SD* = 5.0 %), *t*(60) = 2.27, *p* = .027. In contrast, no difference in accuracy for angry and neutral targets was found in the age task (*p* = .234). Moreover, there was a main effect of age, *F*(1, 59) = 8.96, *MSE* = .004, *p* = .004, as older adults were more accurate (*M* = 98.6 %, *SD* = 1.7 %) than younger adults (*M* = 95.4 %, *SD* = 4.1 %). No further main effects or interactions were observed for accuracy scores for angry vs. neutral faces.

***Reaction times.*** The four-way omnibus ANOVA on repeat trials yielded a significant task × target emotion interaction, *F*(1, 59) = 7.81, *MSE* = 15037, *p* = .007, partial *η^2^* = .12. Follow-up t-test revealed that in the age task, RTs were slower for angry targets (*M* = 783 ms, *SD* = 237 ms) than for neutral targets (*M* = 745 ms, *SD* = 217 ms), *t*(60) = 2.72, *p* = .008. In contrast, no difference in RTs for angry and neutral targets was found in the gender task (*p* = .162). There was also a target emotion × previous emotion interaction, *F*(1, 59) = 5.77, *MSE* = 11867, *p* = .019, partial *η^2^* = .09, which was qualified by a target emotion × previous emotion × age interaction, *F*(1, 59) = 6.37, *MSE* = 11867, *p* = .014, partial *η^2^* = .10. Separate analyses for younger and older adults revealed that younger adults showed a significant target emotion × previous emotion interaction, *F*(1, 28) = 7.93, *MSE* = 17298, *p* = .009, partial *η^2^* = .22, whereas this interaction was not significant in older adults (*p* = .908). Follow-up t-tests showed that younger adults responded faster to neutral targets if the previous emotion was also neutral (*M* = 654 ms, *SD* = 171 ms) rather than angry (*M* = 707 ms, *SD* = 198 ms), *t*(28) = 2.31, *p* = .028. Similarly, they also responded faster to angry targets if the previous emotion was also angry (*M* = 649 ms, *SD* = 120 ms) rather than neutral (*M* = 694 ms, *SD* = 196 ms), *t*(28) = 2.08, *p* = .047. However, the target emotion × previous emotion × age interaction was non-significant (*p* = .159) after the covariates verbal IQ and processing speed were included in the analysis. Similarly, a main effect of age, *F*(1, 59) = 12.22, *MSE* = 274147, *p* = .001, partial *η^2^* = .17. which was driven by slower RTs in older (*M* = 842 ms, *SD* = 208 ms) than in younger adults (*M* = 676 ms, *SD* = 156 ms), became non-significant (*p* = .286) after the covariate processing speed was included in the analysis. No further main effects or interactions were observed for reaction times to happy vs. neutral faces.

## Effects of order of experiments

To check for order effects, all analyses reported in the main paper were repeated with order of experiments as an additional between-subjects factor. For happy vs. neutral faces in Experiment 1, order of experiments affected accuracy as indicated by a task × order of experiments interaction, *F*(1, 58) = 4.50, *MSE* = .005, *p* = .038, partial *η^2^* = .07. Follow-up t-tests revealed that participants who started with Experiment 2 were more accurate in the age task (*M* = 98.4 %, *SD* = 2.9%) than in the emotion task (*M* = 96.4 %, *SD* = 4.1 %), *t*(30) = 2.30, *p* = .029, whereas no such difference in accuracy was observed in those who started with Experiment 1 (*p* = .573). There was also a significant target emotion × order of experiments interaction, *F*(1, 58) = 5.38, *MSE* = .002, *p* = .024, partial *η^2^* = .09. Follow-up t-tests revealed that participants who started with Experiment 1 were more accurate for happy faces (*M* = 98.7 %, *SD* = 2.5%) than for neutral faces (*M* = 97.5 %, *SD* = 3.6 %), *t*(30) = 2.07, *p* = .047, whereas no such difference in accuracy was observed in those who started with Experiment 2 (*p* = .290). No further significant effects were observed for Experiment 1 (all *F*s < 1.5). For happy vs. neutral faces in Experiment 2, order of experiments affected RTs as indicated by a task × order of experiments interaction, *F*(1, 58) = 4.97, *MSE* = 35864, *p* = .030, partial *η^2^* = .08. Follow-up t-tests revealed that participants who started with Experiment 2 were slower in the age task (*M* = 934 ms, *SD* = 267 ms) than in the gender task (*M* = 891 ms, *SD* = 231 ms), *t*(30) = 2.44, *p* = .021, whereas no such difference in RTs was observed in those who started with Experiment 1 (*p* = .503). For angry vs. neutral faces in Experiment 2, a similar result was found. Order of experiments affected RTs as indicated by a marginally significant task × order of experiments interaction, *F*(1, 58) = 3.88, *MSE* = 26776, *p* = .054, partial *η^2^* = .06. Follow-up t-tests revealed that participants who started with Experiment 2 were slower in the age task (*M* = 1083 ms, *SD* = 342 ms) than in the gender task (*M* = 1034 ms, *SD* = 304 ms), *t*(30) = 2.22, *p* = .034, whereas no such difference in RTs was observed in those who started with Experiment 1 (*p* = .624). No further significant effects were observed for Experiment 2 (all *F*s < 2.5).
